# Digital Biomarkers for Well-being Through Exergame Interactions: Exploratory Study

**DOI:** 10.2196/34768

**Published:** 2022-09-13

**Authors:** Despoina Petsani, Evdokimos Konstantinidis, Aikaterini-Marina Katsouli, Vasiliki Zilidou, Sofia B Dias, Leontios Hadjileontiadis, Panagiotis Bamidis

**Affiliations:** 1 Medical Physics and Digital Innovation Laboratory Faculty of Health Sciences, School of Medicine Aristotle University of Thessaloniki Thessaloniki Greece; 2 Centro Interdisciplinar de Estudo da Performance Humana, Faculdade de Motricidade Humana Universidade de Lisboa Lisbon Portugal; 3 Khalifa University of Science and Technology Abu Dhabi United Arab Emirates; 4 Electrical and Computer Engineering Aristotle University of Thessaloniki Thessaloniki Greece

**Keywords:** serious games, machine learning, physical well-being, cognitive well-being

## Abstract

**Background:**

Ecologically valid evaluations of patient states or well-being by means of new technologies is a key issue in contemporary research in health and well-being of the aging population. The in-game metrics generated from the interaction of users with serious games (SG) can potentially be used to predict or characterize a user’s state of health and well-being. There is currently an increasing body of research that investigates the use of measures of interaction with games as digital biomarkers for health and well-being.

**Objective:**

The aim of this paper is to predict well-being digital biomarkers from data collected during interactions with SG, using the values of standard clinical assessment tests as ground truth.

**Methods:**

The data set was gathered during the interaction with patients with Parkinson disease with the webFitForAll exergame platform, an SG engine designed to promote physical activity among older adults, patients, and vulnerable populations. The collected data, referred to as in-game metrics, represent the body movements captured by a 3D sensor camera and translated into game analytics. Standard clinical tests gathered before and after the long-term interaction with exergames (preintervention test vs postintervention test) were used to provide user baselines.

**Results:**

Our results showed that in-game metrics can effectively categorize participants into groups of different cognitive and physical states. Different in-game metrics have higher descriptive values for specific tests and can be used to predict the value range for these tests.

**Conclusions:**

Our results provide encouraging evidence for the value of in-game metrics as digital biomarkers and can boost the analysis of improving in-game metrics to obtain more detailed results.

## Introduction

### Background and Rationale

Serious games (SG) for health are games that aim to provide additional value for players other than mere entertainment and specifically deal with aspects of physical, mental, and social well-being [1], following the World Health Organization’s definition for health [2]. Most findings suggest that SG for health are effective interventions for increasing older people’s mental and physical health and well-being, but there are strong variations in the outcomes and measures used to demonstrate impact [3,4] or assess users during gameplay.

A regularly used method for evaluating the impact of SG interventions is the use of external questionnaires for each participant, one before playing (before the test) and another after going through a series of playing sessions (after the test). This methodology is widely accepted in the health and well-being domain to evaluate the effectiveness of SG [5] but fails to provide more detailed and reliable information [6] on user’s state of health and well-being. Furthermore, the collection of ex situ data such as before the test and after the test requires human resources and is opposed to a more natural experience [7], as they are usually collected in laboratory or clinical settings by clinical experts. Questionnaires, interviews, or test batteries are often used as assessment tools, and when administered, can cause stress to the interviewee, threatening accuracy and ecological validity [8]. On the contrary, the fact that SG can be administered in any setting and in an enjoyable way accounts for an ecologically valid environment where diagnostic processes could become unobtrusive [9]. Thus, the efficiency of using SG for evaluating the state of health and well-being of players should be further investigated, as it is one of the less studied subjects in SG research [10].

Digital SG enable in situ data capturing, which can reveal new insights, except from the usual retrospective analysis of the intervention results. The term *in-game metrics* is usually used to describe in situ data that are collected during the interaction of the user with the SG. In-game metrics can range from the time required to perform a task in the game to a complicated calculation of a score or lower-level data monitoring user interaction. In-game metrics can be very diverse, as there are currently no standards or guidelines of what data should be collected and for what purpose [11]. In-game metrics can be used to adapt to difficulty [12,13], monitor a user’s behavior [14], and evaluate learning progress [15]. Understanding and exploiting in-game metrics is challenging, and the creation of new methods for interpreting in-game metrics into meaningful insights can strengthen SG research and effectiveness [16,17].

The in-game metrics that are generated can be a rich source of information and insights that can potentially be used to predict or characterize a user’s state of health and well-being. The study by Regan et al [18] supports that the data generated during the gameplay, namely the in-game metrics, are promising digital biomarkers for mental health, whereas the study by Staiano and Calvert [19] also argues for the potential of SG to assess physical health. The identification of deviations from the norm in a gameplay or the correlation of in-game metrics with ex situ clinical data can be indicators of mental or physical decline. The reliability and validity of in-game metrics should be further investigated to specify at what extent they can capture changes in health and well-being within research studies. This proof will strengthen the value of in-game metrics as efficient, unobtrusive, and comprehensive research tools to measure participants’ health.

The current work is part of an extended, holistic body of work, the Long Lasting Memories Care (NCT02313935), concerning SG for the physical and cognitive improvement of older adults and other vulnerable populations. The exergame platform of Long Lasting Memories Care has proven to significantly improve strength, flexibility, endurance, and balance in older adults [20]. Furthermore, a previous study conducted by Konstantinidis et al [21] demonstrated the value of in-game metrics generated from body movement interactions with the exergame for detecting cognitive decline, and the study by Anagnostopoulou et al [22] demonstrated evidence of improving the functional architecture of the brain in adults with Down syndrome.

### Related Work

SG have already been used for modeling or characterizing user behavior regardless of health outcomes. The study by Alonso-Fernández et al [23] used the metrics collected during gameplay to predict posttest outcomes using machine-learning algorithms. The study by Loh et al [24] examined the course of actions of players and used several similarity measures to compare players, aiming to differentiate them efficiently and create gaming profiles (distinguishing among fulfillers, explorers, and quitters) in SG. This indicates the potential of using in-game metrics to model different outcomes, depending on what needs to be evaluated.

On the health assessment front, the evaluation and validation of in-game metrics as assessment tools are performed either in comparison with a clinical diagnosis or a validated assessment test [25]. Cognitive measures in game-like interfaces can contribute to the early detection of neurological disease [26]. The study by Bang et al [27] used an index calculated from in-game metrics to detect children with heterogeneous developmental disabilities, whereas the study by Kim et al [28] evaluated the use of kinetic variables from the interactions with an SG as a digital biomarker for developmental disabilities.

SG targeting the improvement of the physical capacity of the player, as the one addressed in this study, are called exergames and can open up a new category of outcome assessment. Exergames are a promising tool for measuring and assessing unobtrusively physical health [19,21] and focus mainly on fall risk assessment by correlating typical in-game metrics of exergames, such as movement time and response time, with a test battery or standardized assessment tests of fall risk [29]. The study by Aguilar et al [30] assessed the effect of 6 weeks of unsupervised home-based SG intervention on dynamic postural control. They used generalized linear models and classification algorithms to estimate the probability that the body movements recorded by Kinect belonged to a participant older than 60 years, and the objective was to distinguish between younger and older participants. However, no further insights were provided regarding the well-being state of the participants. The study by Pirovano et al [25] developed an SG solution to support rehabilitation at home. Their solution combined fuzzy-based monitoring and in-game adaptation to capture the knowledge of the clinician and provide real-time feedback during exercise. This feedback was used for adaptation of the gameplay but not for assessment, although it might have the potential to capture insights for the user movement and rehabilitation process.

Moreover, a review of existing literature has shown that there is a strong interest from the research community in the use of SG, and especially in-game metrics, as psychometric tools and indicators [31]. The study by Valladares-Rodríguez et al [31] identified research issues related to the development of SG for use in neuropsychological evaluation, proving its potential as an alternative to conventional neuropsychological examinations. However, it is pointed out that more research is needed on their reliability and validity for their application in daily clinical practice [32]. In addition, it is necessary to address the risk of investing in technical features that could potentially affect the reliability of the game. For example, to make it so attractive that, during the interaction, the game provokes the respective feature that is called to measure, thus intertwining the purpose of enhancing a feature with that of its measurement [33]. At the same time, they provide many opportunities to enhance the reliability of evaluation processes. In-game metrics can provide information associated not only with the performance outcome of a specific test but also with the processes during the test.

### Study Objectives

This study investigated the possibility of using in-game metrics from exergame interactions as digital biomarkers for well-being. The digital biomarkers investigated are produced from in-game metrics analysis and aim to support the creation of health and well-being profile groups, as well as assess the physical and cognitive state of a user from gameplay without ex situ data. The in-game metrics collected during the interaction of patients with Parkinson disease with the webFitForAll platform [34,35] were used as a case study to validate the study objectives.

## Methods

### Overview

The main analysis included the clustering of participants using the neuropsychological and physical clinical assessment tests that define the ground truth. The clustering was evaluated to select the best grouping of participants. A classification method was used to predict the group to which each participant belonged, using in-game metrics as features. The correlation of each in-game metric with each clinical assessment test was examined to identify those that had a higher separation value. The methodology followed does not rely on the metric choice or any prior knowledge of in-game metrics. We are trying to be metric agnostic, meaning that we are trying to find an explanation for how the data correlations with the clinical assessment tests are ruled out.

### webFitForAll Platform

This study analyzed the data captured during the interaction with the webFitForAll platform [20]. webFitForAll is a web-based platform that provides SG for exercising that are specially designed and tested for older adults and vulnerable populations. The users interact with the game using body movements captured with 3D depth sensor controllers. There are additional games on the platform that are controlled by other types of user interfaces, such as touch screens and voice control. This study focuses on the exergames of the platform, which are controlled by body movements to obtain unified results. The games used in this study were as follows: (1) fishing, (2) kinematic orchestra, (3) picking citrus fruits, and (4) retraining in eating behavior [36]. The games were designed to address specific gait, balance, and exercising needs of patients with for Parkinson disease using a participatory design methodology [36].

In the fishing game, the user’s body posture is translated into the direction and velocity of a digital boat. Leaning forward will result in a forward movement of the boat with acceleration proportional to the body inclination. The goal is to collect as many fish as they can, in a specific period, by driving the boat toward the fish, avoiding at the same time the obstacles (rocks and sharks) and counterbalance the wind that might alter their direction [37].

In the kinematic orchestra game, the user tries to associate a specific group of notes with hand gestures such as moving the left or right hand up, down, or cyclical, or lower body movements such as raising the right or left leg. The note groups are presented sequentially, and the user is given a specific period to recognize the group, match it with a specific movement, and perform the movement [37].

In the picking citrus fruits game, the user navigates in a virtual environment by walking on spot. Specific instructions on picking and putting down fruits are presented on the screen regarding the sequence of actions the user needs to perform. The user is picking fruits by moving either the left or right hand. The climbing is simulated by walking on spot. The goal is to pick as many fruits as possible in a specific period [37].

In the retraining of the eating behavior game, the user faces the screen, either seated or standing, and pretends to hold a spoon or fork. An avatar is presented on the screen and shows the correct frequency of movement. The user moves their hand to imitate the movement of bringing the spoon in the mouth. The game monitors the movement and correlates it with the correct movement presented on the screen. Every time the user maintains the correct frequency, they earn a point. The game lasted for a specific period [37].

### Data Set

#### In-Game Metrics

During participant interaction with the games, the system manually captures metrics that are representative and can provide insights for each game. For every game, different in-game metrics correspond to different measures. Each in-game metric is described in Textbox 1.

**In-game metrics.**

**Fishing**

- Score: this in-game metric captures how many fish the user collects during a specific time of gameplay. If a user runs into obstacles (sharks or rocks), the score is reduced. Therefore, the score is a combination of moving toward the goal (fish) and moving away from obstacles (sharks and rocks).
- Goal time: it is the duration between the time point when the target fish is presented in the screen until the time point that the user captures the fish. This duration was calculated for each fish caught by the user. For each session (Si), the in-game metric is a sequence of values representing the time for each reached goal.

**Kinematic orchestra**

- Score: this in-game metric measures the number of movements correctly performed within a limited period. The game recognizes whether the user has performed the correct movement based on the matching between note groups and movements, and that the user was able to react quickly enough.
- Goal time: this is the duration between the time point when the target note group is presented on the screen until the time point when the user performs the right movement. This duration was calculated only for movements performed correctly. For each session (Si), the in-game metric is a sequence of values representing the time for each reached goal.

**Picking citrus fruits**

- Score: this in-game metric captures the number of fruits the user collects during a specific time of gameplay.
- Goal time: this is the duration between the time point when the targeted fruit becomes highlighted and the time point that the user “catches” the fruit. This duration is calculated for each fruit that the user catches. For each session (Si), the in-game metric is a sequence of values representing the time for each reached goal.

**Retraining of eating behavior**

- Score: this in-game metric captures the number of correct movements that the user performs during a specific time of gameplay. Correct movement is considered to occur almost simultaneously with the avatar presented on the screen. The game allows a specific time window that is sufficiently small to consider that the movement is performed with the same frequency.
- Goal time: this is the difference between the time point at which the avatar performs the movement and the time at which the user performs the movement. For each session (Si), the in-game metric is a sequence of values representing the time for each reached goal.

All data collected during the sessions were stored in an SQL database, pseudoanonymized, and password-protected for each participant. The data set was retrieved from the database for offline analysis and fully anonymized for this study.

#### Clinical Neuropsychological and Physical Assessment Tests

Before and after every sequence of intervention, in a time window of no longer than 1-week, clinical neuropsychological and physical assessment tests were administered to each participant. These tests were performed by professionals, psychologists, and physical educators. They were considered the ground truth for each participant’s cognitive and physical state before and after the intervention. The administered tests were carefully selected to depict all the domains that were influenced by the SG, as well as the domains that were mostly influenced by Parkinson disease. The selected tests assess various levels of physical status as well as cognitive impairment and meet specific criteria such as validity, reliability, and objectivity. The administered tests were the Single-Leg-Stance Test [38], Berg Balance Scale [39], Short Physical Performance Battery [40], Community Balance and Mobility Scale (CB&M), Senior Fitness Test (Fullerton Fitness Test) [41], BMI [42], Performance-oriented Mobility Assessment [43], 10 Meter walk, Instrumental Activities of Daily Living Scale [44], 8-item Parkinson’s Disease Questionnaire [45], Fall Risk Assessment [46], Dementia Rating Scale [47], and Symbol Digit Modalities Test (Symbol) [48].

### Protocol

**Figure 1:**
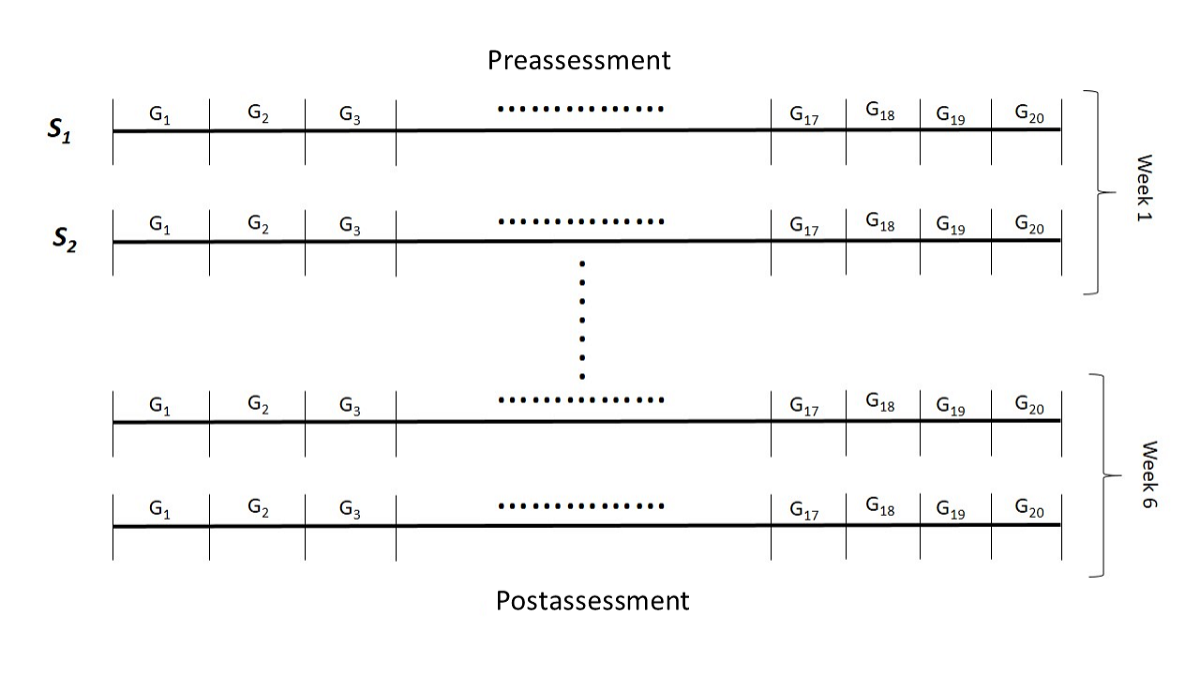
Visualization of a participant study. G1 represents the sequence of games.

Every participant enrolled in the study had to attend at least 16 sessions not to be considered a dropout. Every session (Si) comprised the same sequence of games (Gi), including 20 games in total. The sessions were performed twice a week on predefined days. A participant could be reenrolled in a study for a follow-up series of interventions. However, the data from follow-up sessions were not considered a unified participant study if they were performed in a period of more than 1 week from the last session. Figure 1 presents a visualization of the protocol performed by each participant. A participant study was defined as one in which data were collected from sessions performed continuously, composed the same game sequence in every session, and came from a single participant.

From the aforementioned sequence of games (Gi), 4 were used for the analysis (fishing, kinematic orchestra, picking citrus fruits, and retraining in eating behavior). These correspond to exergames and are captured in a unified manner using depth sensor cameras. These are the ones that require body movement interactions with the game and were suggested by health care professionals as the most indicative for capturing the patient’s condition and the most commonly appearing Parkinson disease symptoms. The protocol also included preclinical and postclinical neuropsychological and physical assessments using standardized questionnaires, as presented in the previous section. The participants were also engaged in 1 testing session before the actual intervention period to familiarize themselves with the games and eliminate the effect of nonrepresentative game measures owing to misunderstandings in the first intervention.

### Participants

The experimental data set that was used for testing the approach in this study consists of gaming sessions that took place within the i-Prognosis H2020 project [49] within day care centers of the “Northern Greece Association of Parkinson’s Disease Patients and Friends.” A total of 13 participants, all diagnosed with Parkinson disease, with a mean age of 64.5 (SD 9.3) years, participated in the study protocol. All participants signed an informed consent form, and no financial incentives were provided to them. The participants interacted with the webFitForAll platform twice a week in sessions of 60 minutes each. Each participant performed a mean number of 25.9 (SD 5.4) sessions. Clinical assessment tests were administered within a week before entering the study (before the assessment) and within 1 week after completing the series of intervention sessions (after the assessment).

### Analysis Methodology

#### Preprocess

**Figure 2:**
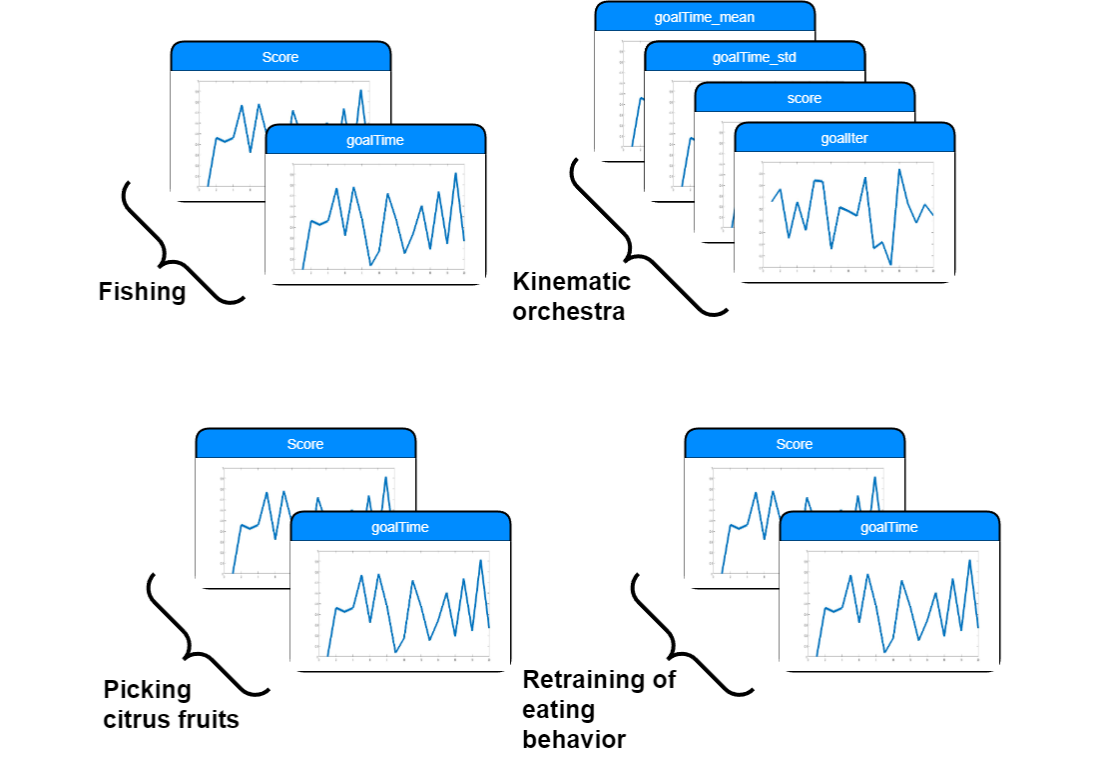
Visual representation of mock data set representing a participant study.

The mean and SD values were calculated for each in-game metric per session. The result for each user is a time series for each in-game metric, with every point in the time series representing the value for 1 session. Each user played various games and different in-game metrics were captured for each game. A visual representation of the data set collected for each user is shown in Figure 2.

After collecting all the data, the outliers for each in-game metric were found and removed using the IQR based on the quartile method for the detection of outliers [50]. IQR is the range between the median of the upper and lower halves of the data. A total of 4 quartiles were computed for each in-game metric series, and the IQR was calculated as IQR=Q3-Q1. A point in an in-game metric was considered an outlier if its value exceeded the value of 1.5×IQR and was then removed from the data set. In addition, we removed extreme values that were produced when the human skeleton was not detected properly because of the hardware resolution and monitoring conditions. Some extreme values were also produced by software bugs that were subsequently identified using the log files of the system.

The next step was to extract the features from each in-game metric time series. The mean and SD values of the time series were selected as features. The first 5 values of each in-game metric time series, henceforth referred to as PRE in-game metric data, were calculated for each in-game metric feature. The first 5 sessions were considered to better reflect the physical and mental state of the participants before the beginning of the intervention, absorbing any artifacts from the games learning procedure. Considering only 1 game point in the analysis can affect the reliability of results.

#### Clustering

A set of neuropsychological and physical assessment tests was used as the ground truth for separating participants into groups with better or worse physical and cognitive states using clustering analysis. The hierarchical agglomerative clustering (HAC) algorithm was used for clustering. In HAC, each observation is initially considered a separate class. The algorithm chooses clusters that are most similar to each other and merges them into 1 cluster, which continues until all objects are merged into a single cluster.

#### Classification

A classification method was used to predict the group each participant belongs to using, as features, the in-game metrics. Each feature set consists of all in-game metric values following the feature extraction procedure described earlier. The decision tree classifier was selected as the classification method because the internal decision-making logic is clear and transparent, which is not the case for black box-type algorithms such as neural networks. Decision tree classifiers are fast to train and allow the capture of descriptive decision-making knowledge that can help us interpret results. The decision rules produced by the decision tree classifier can also be exploited in the design and interpretation of other in-game metrics. The selection measure used in this study is the Gini index, which measures the probability that a specific variable is incorrectly classified when its class is randomly chosen.

The leave-one-out method was used to mitigate the low volume of data. In this method, the data set was first separated into m number of data sets each containing 1 feature vector for 1 participant. In each iteration, 1 feature vector was kept for testing, whereas the other m-1 feature vectors were used for training the model. When all feature vectors have been used 1 time for testing, the process is complete. Thus, for every feature vector, there is an assigned class, which is the predicted class.

#### Analyzing Each In-Game Metric Separately

The aim of this step is to identify the contribution of each in-game metric to the prediction of the well-being status that corresponds to each assessment test. To do so, a feature vector containing the mean value of all sessions for each in-game metric was calculated for each user. In addition, the clinical assessment test feature vector was calculated, which corresponded to the different states of well-being. This vector consisted of the mean value for each preassessment and postassessment test. The Pearson correlation coefficient between the in-game metrics and clinical assessment tests was calculated. The results were considered to specify which in-game metrics could predict which assessment test. Metrics with a strong correlation (high Pearson correlation coefficients) were selected for further analysis.

Representing each time series with a single value lacks information about the progression of values and how they change over time, which might be present in the whole time series. To avoid this and consider the evolution of each participant throughout the sessions, the dynamic time warping (DTW) [51] method was used. DTW is a method used in time series analysis to measure the similarity of 2 time series, even if they have differences in speed. For example, 2 time series can have the same form, but vary in time length and data points. The values that demonstrated a strong correlation in the previously described step were used to calculate the distance matrix using the DTW method. This distance matrix was then used as the input for HAC analysis.

### Ethics Approval

This study was approved by the School of Medicine Bioethics Committee (protocol number 4.123, 17/7/2019).

## Results

First, we present the results of the general analysis considering all games and their in-game metrics, as well as all clinical assessment tests. Then, the results presented focus on the picking citrus fruits score in-game metric, which has the highest predictive value according to the general results.

### Clustering

The HAC clustering algorithm using the Euclidean distance metric and ward linkage resulted in the following groups:

- Group 0: p#2, p#6, p#8, p#9
- Group 1: p#0, p#1, p#3, p#4, p#5, p#7, p#10, p#11, p#12

**Figure 3:**
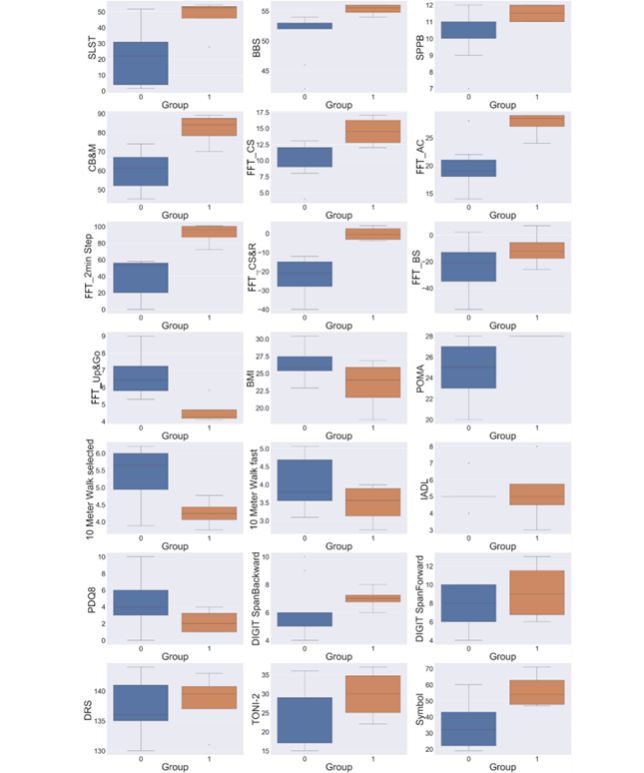
Clinical assessment test values for the 2 different groups formed after clustering. BBS: Berg Balance Scale; CB&M: Community Balance and Mobility; FFT: Fullerton Fitness Test; FFT_AC: Fullerton Fitness Test arm curl; FFT_BS: Fullerton Fitness Test back scratch; IADL: Instrumental Activities of Daily Living; PDQ-8: 8-item Parkinson’s Disease Questionnaire; POMA: Performance-Oriented Mobility Assessment; SPPB: Short Physical Performance Battery; TONI-2: Test of Nonverbal Intelligence, 2nd edition.

Figure 3 presents boxplots of the clinical assessment tests for the 2 different groups of participants that were formed after clustering. In most cases, the 2 formed groups demonstrated a large difference in the distribution of values, which is an indication of good intercluster differences.

In general, group 0 scored lower range of values compared with group 1 in most tests. The image is different in Fullerton Fitness Test (FFT) foot up and go, BMI, and 10 Meter Walk, but these are tests that show higher values indicate lower capacity. Thus, we can safely conclude that group 0 included participants with lower physical and cognitive capacities, whereas group 1 included participants with higher physical and cognitive capacities.

### Classification

The decision tree classifier (criterion=Gini) was evaluated using the leave-one-out method. The goal was to predict if a user belongs to group 0, indicating lower physical and cognitive capacity or in group 1, indicating higher physical and cognitive capacity using information coming only from in-game metrics. The mean values of the initial measurements (PRE) of the in-game metrics were used as features for the prediction. A high classification accuracy (0.846) shows the capacity of in-game metrics to distinguish users with comparable discriminant values to the clinical assessment tests. The confusion matrix and evaluation metrics are presented in Table 1.

**Table 1:** Confusion matrix and results from classification.

True label	Predicted label^a^	
	Zero	One
One	True negative 8^b^ 61.54%	False Positive 1 7.69%
Zero	False negative 1 7.69%	True Positive 3 23.08%

^a^Accuracy 0.846; recall 0.75; precision 0.75; *F*_1_-score=0.75.

^b^The absolute numbers are the instances that were true negative, false positive, etc, and the percentage of instances to the total number of instances.

The model also demonstrated high recall, precision, and *F*_1_-score values, which further supports the sensitivity of the model for detecting participants with both higher and lower levels of physical and cognitive capacity.

Participant p#12 was falsely assigned to group 1 by the classifier, whereas participant p#2 was falsely assigned to group 0.

### Analyzing Each In-Game Metric Separately

**Figure 4:**
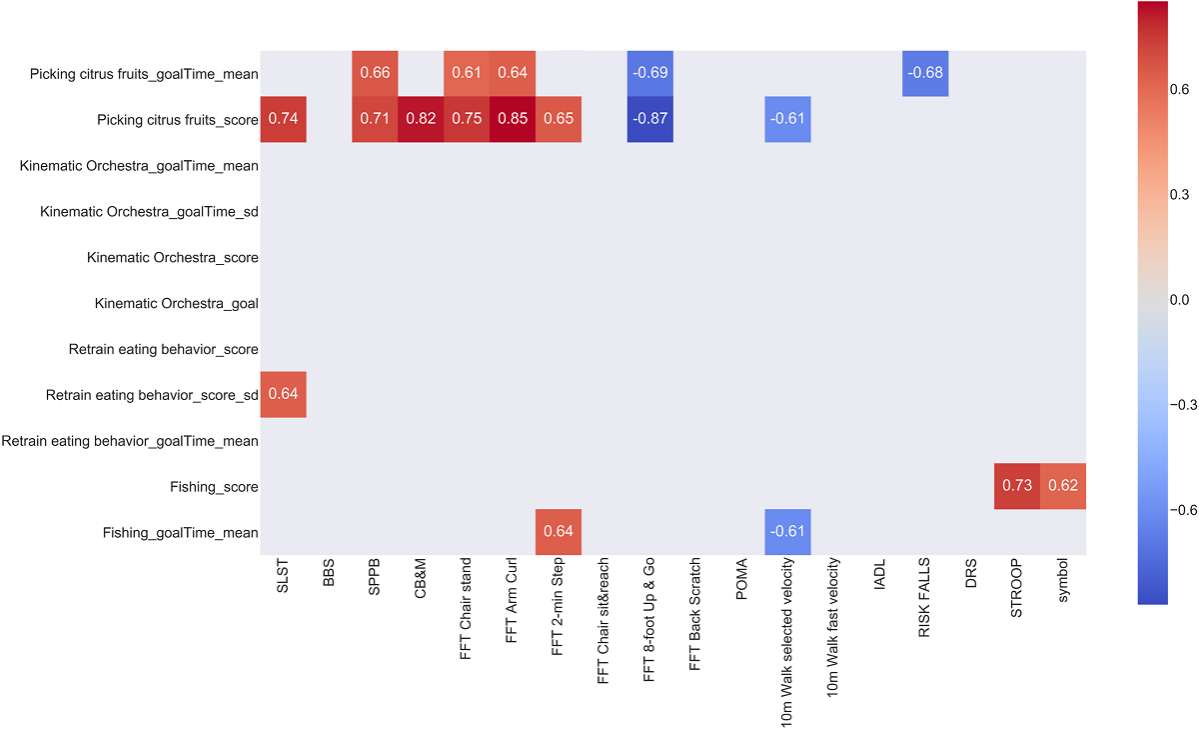
Pearson correlation of in-game metrics series with assessment tests. BBS: Berg Balance Scale; CB&M: Community Balance and Mobility; FFT: Fullerton Fitness Test; IADL: Instrumental Activities of Daily Living; POMA: Performance-Oriented Mobility Assessment; SPPB: Short Physical Performance Battery.

The Pearson correlation coefficient of the in-game metrics series with the clinical assessment tests is presented in Figure 4. Only the correlations with absolute values higher than 0.6 are presented. In-game metrics with a high correlation with specific test values can be used to build a model that predicts the exact value of the assessment test and not just a general class that separates the group of participants into higher and lower physical and cognitive capacity.

From Figure 4, we conclude that the picking citrus fruits score in-game metric has a higher degree of correlation with most clinical assessment tests. Thus, this in-game metric is the most reliable for further investigation of its correlation with each clinical assessment test separately. Although the picking citrus fruits score has a strong correlation with various assessment tests, this in-game metric alone cannot be used to separate participants into 2 categories based on their physical and cognitive status.

The time series for each participant for the picking citrus fruits score in-game metric was used to calculate the distance matrix using the DTW method. This distance matrix, using HAC with complete linkage, resulted in 3 clusters based on their performance in the specific metric (picking citrus fruits score):

- Group 1: p#9, p#6, p#8
- Group 2: p#3, p#5
- Group 3: p#7, p#12, p#11, p#0, p#4, p#2, p#1, p#10

**Figure 5:**
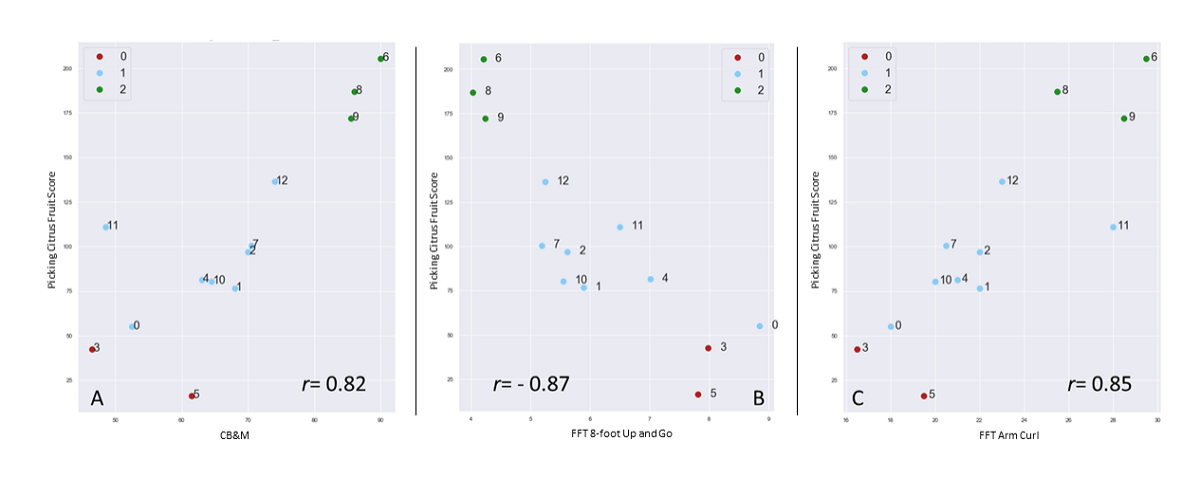
Community Balance and Mobility test versus picking citrus fruits game score (right), Fullerton Fitness Test (FFT) 8-foot up and go test versus picking citrus fruits game score (center), and 10 FFT arm curl test versus picking citrus fruits game score (left). The identified groups are presented with different colors. CB&M: Community Balance and Mobility; FFT: Fullerton Fitness Test.

To further assess the results of clustering, we present the CB&M, the FFT arm curl, and the FFT 8-foot up and go mean prevalues and postvalues in correlation with the mean picking citrus fruits score in-game metric, which are the tests with higher correlation values, as shown in Figure 5. The different identified groups are presented with different colors.

All 3 clinical assessment tests demonstrated a strong correlation with the in-game metric (*r*=0.82, *r*=−0.87, and *r*=0.85) and clear separation of the 3 clusters created using the whole time series. Group 0 corresponds to participants with lower capacity and performance, group 1 corresponds to medium performance, and group 2 includes the best players with higher physical capacity. Participants p#6, p#8, and p#9 were assigned to the clusters that correspond to the “good” participants in both the aggregated study and considering only 1 in-game metric. Participant p#0 was close to the lower capacity group in all cases but was clustered in the medium performance group based on the picking citrus fruits game score that the participant achieved over time. Participant p#0 was assigned to the higher-capacity group in the clustering, which shows the importance of considering a combination of in-game metrics for the assessment. It is important to point out that the 3 tests with higher correlation values assess physical capacity, which indicates that it is important to include other in-game metrics to assess cognitive capacity as well.

## Discussion

### Principal Findings

This study explored the possibility of using in-game metrics as digital biomarkers to gain meaningful insights into participants’ physical and cognitive states. The analysis was performed in two different steps: (1) considering the whole data set of clinical assessment tests to compute a baseline and then classifying participants based on in-game metrics and (2) evaluating the value of a single in-game metric to predict the participants’ physical state.

The analysis returned very promising results, achieving an 85% accuracy in distinguishing the 2 groups of participants based on their performance in the preneuropsychological and physical assessment tests (group 0 indicates lower physical and cognitive capacity, whereas group 1 indicates higher physical and cognitive capacity). Considering the classification of the participants from in-game metrics, a professional can obtain an idea for the participant profile based on the characteristics of each group in the corresponding cognitive and physical assessment (Figure 4). The proposed methodology can be generalized for further use in other types of SG and in-game metrics using different standard tests as the ground truth for each user, as it is metric agnostic.

Although the use of a combination of in-game metrics as a digital biomarker for characterizing well-being returned promising results, the separate use of each in-game metric can provide more detailed information for specific physical and cognitive capacities. Picking citrus fruits score in-game metric achieved a high correlation with multiple physical assessments and can be further considered as a digital biomarker for characterizing physical status. The picking citrus fruits game that demonstrated the highest descriptive value, targets improvement of motor skills, synchronization, and balance skills [52]. It shows high correlations with physical tests, such as FFT and CB&M, which provide insights into the physical state of the person and the movement of the lower body. Achieving a higher score in the picking citrus fruits game can be explained by the fact that a player with higher stability and strength in the lower body has higher confidence and performs the exercise with precision and speed. This translates to higher clinical assessment scores.

### Insights From Professionals

A total of 4 professional experts in the health and well-being domain assisted the participants in their day-to-day interactions with the games and performed clinical assessments. The professionals evaluated the participants’ performance, overall capacity, and value of the proposed games. Professionals considered the fishing and picking citrus fruits games as games that can more effectively separate participants into 2 groups. This supports the results of this study, as the 2 games yielded higher correlations with the ground truth assessment test.

Participants p#6, p#8, and p#9 were considered as the players with the higher capacity and performance by professionals, which is also reflected in the results of the current analysis. These players were always classified in the group of “better” cognitive and physical state. The professionals working with p#12 commented that the participant was not very concentrated during the sessions and used to talk frequently. This may explain the false classification results and incoherent outcomes between the 2 analyses. Participant p#0 was assigned to group 1 based on the picking citrus fruits in-game metric, although it had low scores in physical assessment tests. Professionals working with the participant commented that besides the low physical capacity, the participant had tried a lot and showed great progress. This explains why the DTW distance score was lower for participants in group 1, indicating a tendency to improve, and hence, assign the participant to that group.

In the case of a system with both interventional and assessment capabilities, such as webFitForAll, the value for the participants could be 2-fold. First, delay in cognitive decline onset as physical exercise is a preventive intervention for cognitive decline [53]. Second, early detection of cognitive and physical decline symptoms would provide the opportunity for the early administration of available treatments when interventions are more effective [34,54].

The presented methodology could be useful in categorizing players with very high and poor performance. The transition from one group to another throughout the course of the intervention sessions can be an alarm for further evaluation. The first set of sessions can also be exploited to collect data to guide the design of individualized interventions and specify areas of difficulty and behavioral response patterns to the skills being tested.

### Comparison With Previous Studies

There is an increasing body of research that investigates the complementarity of digital tools for measuring health, well-being, and clinical outcomes, along with existing methods. Similar studies have investigated the use of 3D depth sensor cameras (Microsoft Kinect) in game design to identify patients with spinal muscular atrophy and healthy controls [55]. Similar to our results, they identified some digital biomarkers that can detect differences (eg, hand velocity), whereas other minor differences in functioning cannot be detected. The study by Gielis et al [56] used a combination of data produced by a casual card game as digital biomarkers to distinguish mild cognitive impairment from healthy participants. Their results were similar to ours (accuracy 0.792), but a direct comparison of the 2 studies could not be performed. However, both studies focused on the suitability of analyzing in-game metrics for characterizing participants’ health and well-being.

In-game metrics cannot substitute for the use of clinical neuropsychological and physical evaluation, but can be used as an auxiliary method of amplifying and cross-referencing results from the traditional method of evaluation. Beyond SG, recent studies present computerized forms of cognitive assessment tests where the results are compared against the corresponding pen and paper tests, which exhibit a strong correlation [26]. These game-like screening tests focus mainly on the assessment of the player and not on the intervention, thereby confusing the term “game” with the term “computerized test.” In addition, a number of computerized cognitive screening tests have been evaluated with respect to their sensitivity and specificity with high accuracy [57-59]. Each SG consists of a multifactorial stimulus and improvement features, whereas assessment tests provide a structured evaluation method that specifically targets 1 factor. In-game metrics can provide detailed and rich data on processes and the progress of the participant, which can be used for evaluation over time and individually for each participant.

Although digital biomarkers from SG show significant discriminative value, a more in-depth contextual analysis is required per case. A better understanding of each target group’s capabilities and particularities can lead to further adaptation and selection of measures.

### Limitations

Some of the limitations of the study are the small number of participants (N=13) and individual factors, such as partial symptomatology and the course of Parkinson disease, that significantly differentiated the conditions for each of the participants and because of the small sample size as variables bear great weight in the influence of the results. Owing to the small sample size, the above method of dividing into groups of users with good performance and those with poor performance is not very sensitive in locating players with more specific performance profiles and unstable performance.

In addition, most games were played with the help of a facilitator. The facilitator played a supporting role, but sometimes their comments could bias the in-game metric results. This research methodology could be applied to larger samples and provide safer results, as the influence of individual endogenous and environmental factors would be reduced. Factors such as hesitation to participate, stress, or preexisting depression can be assessed at the beginning of the intervention and can be used to weigh the individual results as they can affect the engagement and effort put by each participant.

Finally, the SG were initially designed as interventions and did not aim to assess the participants. However, as their value as screening methods becomes apparent, a redesign in that direction could support their valorization as decision-support tools.

### Conclusions

SG have recently been used as a reach source of unobtrusively captured information about the user that can drive the creation of digital biomarkers for assessing health and well-being. This study explores the use of the webFitForAll platform, which collects in-game metrics from user movement during gameplay, to identify different user profiles compared with a baseline created by clinical assessment tests. The results are promising and can boost the analysis for improving in-game metrics to obtain more detailed results. More in-game metrics can be gathered during the analysis, specifically targeting the prediction of assessment tests.

## Abbreviations

DTW: dynamic time warping
FFT: Fullerton Fitness Test
HAC: hierarchical agglomerative clustering
SG: serious games
